# 2-[6,8-Dibromo-3-(4-hydroxy­cyclo­hexyl)-1,2,3,4-tetra­hydro­quinazolin-2-yl]-6-methoxy­phenol

**DOI:** 10.1107/S1600536809005182

**Published:** 2009-02-18

**Authors:** Zhi-Gang Wang, Rong Wang, Yi Zhang, Feng Zhi, Yi-Lin Yang

**Affiliations:** a Respiratory Department, Third Affiliated Hospital of Soochow University, Changzhou 213003, People’s Republic of China; b Modern Medical Research Center, Third Affiliated Hospital of Soochow University, Changzhou 213003, People’s Republic of China; c Department of Neurosurgery, Third Affiliated Hospital of Soochow University, Changzhou 213003, People’s Republic of China

## Abstract

The title compound, C_21_H_24_Br_2_N_2_O_3_, was synthesized by the condensation reaction of 3-methoxy­salicylaldehyde with 4-(2-amino-3,5-dibromo­benzyl­amino)cyclo­hexa­nol in a methanol solution. The dihedral angle between the two benzene rings is 76.4 (3)°. The cyclo­hexyl ring adopts a chair configuration. There is an intra­molecular O—H⋯N hydrogen bond which affects the solid state conformation of the mol­ecule. The crystal structure is stabilized by inter­molecular O—H⋯O hydrogen bonds, forming chains running along the *b* axis.

## Related literature

For details of the pharmaceutical uses of the closely related compound ambroxol, systematic name 4-(2-amino-3,5-di­bromo­benzyl­amino)cyclo­hexa­nol, see: Felix *et al.* (2008 ▶); Gaida *et al.* (2005 ▶); Lee *et al.* (2004 ▶). For bond length data, see: Allen *et al.* (1987 ▶).
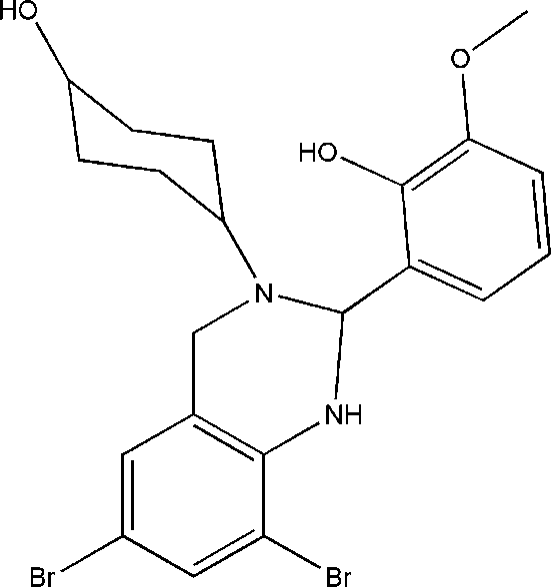


## Experimental

### 

#### Crystal data

C_21_H_24_Br_2_N_2_O_3_
*M* *_r_* = 512.24
Triclinic, 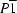

*a* = 8.695 (2) Å
*b* = 11.124 (3) Å
*c* = 12.090 (2) Å
α = 73.870 (3)°
β = 78.226 (3)°
γ = 67.031 (2)°
*V* = 1028.2 (4) Å^3^
*Z* = 2
Mo *K*α radiation
μ = 3.97 mm^−1^
*T* = 298 K
0.30 × 0.30 × 0.30 mm

#### Data collection

Bruker SMART CCD area detector diffractometer
Absorption correction: multi-scan (*SADABS*; Sheldrick, 1996 ▶) *T*_min_ = 0.382, *T*_max_ = 0.382 (expected range = 0.304–0.304)
8514 measured reflections
4305 independent reflections
3035 reflections with *I* > 2σ(*I*)
*R*_int_ = 0.032

#### Refinement

*R*[*F* ^2^ > 2σ(*F* ^2^)] = 0.042
*wR*(*F* ^2^) = 0.096
*S* = 1.02
4305 reflections
256 parameters
H-atom parameters constrained
Δρ_max_ = 0.51 e Å^−3^
Δρ_min_ = −0.41 e Å^−3^

### 

Data collection: *SMART* (Bruker, 2002 ▶); cell refinement: *SAINT* (Bruker, 2002 ▶); data reduction: *SAINT*; program(s) used to solve structure: *SHELXS97* (Sheldrick, 2008 ▶); program(s) used to refine structure: *SHELXL97* (Sheldrick, 2008 ▶); molecular graphics: *SHELXTL* (Sheldrick, 2008 ▶); software used to prepare material for publication: *SHELXTL*.

## Supplementary Material

Crystal structure: contains datablocks global, I. DOI: 10.1107/S1600536809005182/sj2575sup1.cif

Structure factors: contains datablocks I. DOI: 10.1107/S1600536809005182/sj2575Isup2.hkl

Additional supplementary materials:  crystallographic information; 3D view; checkCIF report

## Figures and Tables

**Table 1:** Hydrogen-bond geometry (Å, °)

*D*—H⋯*A*	*D*—H	H⋯*A*	*D*⋯*A*	*D*—H⋯*A*
O2—H2⋯N2	0.82	1.89	2.614 (3)	147
O1—H1⋯O3^i^	0.82	2.41	3.048 (4)	135
O1—H1⋯O2^i^	0.82	2.13	2.897 (4)	155

Symmetry code: (i) 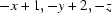.

**Fig. 1.**
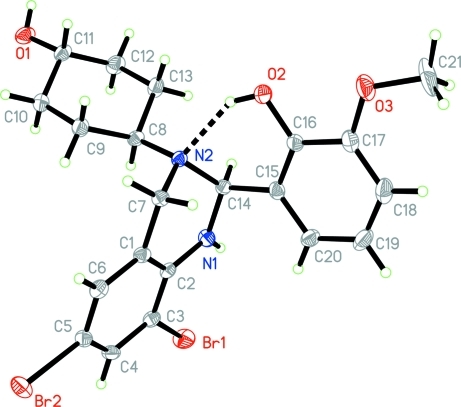
The structure of (I) at the 30% probability level. The intramolecular O–H···N hydrogen bond is shown as a dashed line.

**Fig. 2.**
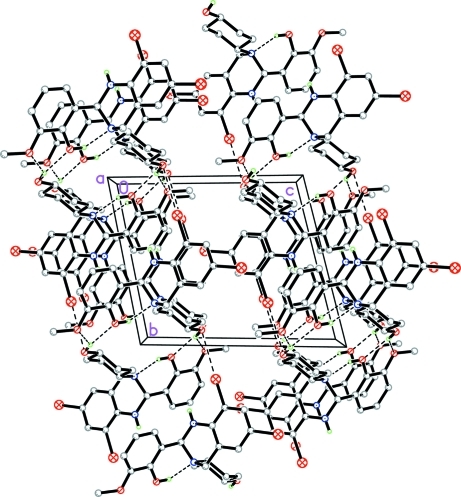
Molecular packing of (I), viewed along the a axis. Intermolecular hydrogen bonds are shown as dashed lines.

## supplementary crystallographic information

### Comment

Ambroxol, 4-(2-amino-3,5-dibromobenzylamino)cyclohexanol, is an expectorant agent which leads to bronchial secretion due to its mucolytic properties (Felix *et al.*, 2008; Gaida *et al.*, 2005; Lee *et al.*, 2004). In this paper, the crystal structure of the new title compound, (I), derived from the condensation reaction of 3-methoxysalicylaldehyde with 4-(2-amino-3,5-dibromobenzylamino)cyclohexanol in a methanol solution, is reported.

In (I), Fig. 1, the dihedral angle between the two benzene rings is 76.4 (3)°. The cyclohexyl ring adopts a chair configuration. All the bond lengths are within normal ranges (Allen *et al.*, 1987). There is an intramolecular O2—H2···N2 hydrogen bond which affects the solid state conformation of the molecule. The crystal structure is stabilized by intermolecular O—H···O hydrogen bonds (Table 1), forming chains running along the *b* axis (Fig. 2).

### Experimental

3-Methoxysalicylaldehyde (1.0 mol, 152.1 mg) and 4-(2-amino-3,5-dibromobenzylamino)cyclohexanol (1.0 mmol, 378.1 mg) were dissolved in a methanol solution (10 ml). The mixture was stirred at room temperature to give a clear colorless solution. Crystals of the title compound were formed by gradual evaporation of the solvent for a week at room temperature.

### Refinement

H atoms were constrained to ideal geometries, with C–H = 0.93–0.97 Å, O–H = 0.82 Å, and with *U*_iso_(H) set to 1.2*U*_eq_(C) and 1.5*U*_eq_(O and C21).

### Crystal data

**Table:**

C_21_H_24_Br_2_N_2_O_3_	*Z* = 2
*M**_r_* = 512.24	*F*(000) = 516
Triclinic, *P*1	*D*_x_ = 1.655 Mg m^−^^3^
Hall symbol: -P 1	Mo *K*α radiation, λ = 0.71073 Å
*a* = 8.695 (2) Å	Cell parameters from 2079 reflections
*b* = 11.124 (3) Å	θ = 2.3–24.6°
*c* = 12.090 (2) Å	µ = 3.97 mm^−^^1^
α = 73.870 (3)°	*T* = 298 K
β = 78.226 (3)°	Block, colorless
γ = 67.031 (2)°	0.30 × 0.30 × 0.30 mm
*V* = 1028.2 (4) Å^3^	

### Data collection

**Table:**

Bruker SMART CCD area detector diffractometer	4305 independent reflections
Radiation source: fine-focus sealed tube	3035 reflections with *I* > 2σ(*I*)
graphite	*R*_int_ = 0.032
ω scans	θ_max_ = 27.0°, θ_min_ = 1.8°
Absorption correction: multi-scan (*SADABS*; Sheldrick, 1996)	*h* = −11→10
*T*_min_ = 0.382, *T*_max_ = 0.382	*k* = −14→14
8514 measured reflections	*l* = −15→15

### Refinement

**Table:**

Refinement on *F*^2^	Primary atom site location: structure-invariant direct methods
Least-squares matrix: full	Secondary atom site location: difference Fourier map
*R*[*F*^2^ > 2σ(*F*^2^)] = 0.042	Hydrogen site location: inferred from neighbouring sites
*wR*(*F*^2^) = 0.096	H-atom parameters constrained
*S* = 1.02	*w* = 1/[σ^2^(*F*_o_^2^) + (0.0423*P*)^2^ + 0.0168*P*] where *P* = (*F*_o_^2^ + 2*F*_c_^2^)/3
4305 reflections	(Δ/σ)_max_ = 0.001
256 parameters	Δρ_max_ = 0.51 e Å^−^^3^
0 restraints	Δρ_min_ = −0.41 e Å^−^^3^

### Special details

**Table:**

Geometry. All e.s.d.'s (except the e.s.d. in the dihedral angle between two l.s. planes) are estimated using the full covariance matrix. The cell e.s.d.'s are taken into account individually in the estimation of e.s.d.'s in distances, angles and torsion angles; correlations between e.s.d.'s in cell parameters are only used when they are defined by crystal symmetry. An approximate (isotropic) treatment of cell e.s.d.'s is used for estimating e.s.d.'s involving l.s. planes.
Refinement. Refinement of *F*^2^ against ALL reflections. The weighted *R*-factor *wR* and goodness of fit *S* are based on *F*^2^, conventional *R*-factors *R* are based on *F*, with *F* set to zero for negative *F*^2^. The threshold expression of *F*^2^ > σ(*F*^2^) is used only for calculating *R*-factors(gt) *etc*. and is not relevant to the choice of reflections for refinement. *R*-factors based on *F*^2^ are statistically about twice as large as those based on *F*, and *R*- factors based on ALL data will be even larger.

### Fractional atomic coordinates and isotropic or equivalent isotropic displacement parameters (Å^2^)

**Table:**

	*x*	*y*	*z*	*U*_iso_*/*U*_eq_	
Br1	1.17738 (5)	0.24247 (4)	0.32321 (4)	0.04726 (14)	
Br2	1.28346 (5)	0.54977 (4)	0.58131 (3)	0.04402 (14)	
O1	0.2331 (3)	0.9422 (3)	0.3052 (2)	0.0418 (7)	
H1	0.1581	0.9883	0.2635	0.063*	
O2	0.9413 (3)	0.8807 (3)	−0.1082 (2)	0.0430 (7)	
H2	0.8961	0.8692	−0.0416	0.065*	
O3	1.1435 (4)	0.9116 (3)	−0.2940 (2)	0.0510 (7)	
N1	1.0518 (4)	0.5182 (3)	0.1542 (2)	0.0305 (7)	
H1A	1.0527	0.4432	0.1469	0.037*	
N2	0.9133 (3)	0.7617 (3)	0.1103 (2)	0.0249 (6)	
C1	1.1053 (4)	0.6460 (3)	0.2638 (3)	0.0265 (8)	
C2	1.1080 (4)	0.5243 (3)	0.2507 (3)	0.0263 (7)	
C3	1.1674 (4)	0.4110 (3)	0.3381 (3)	0.0287 (8)	
C4	1.2199 (4)	0.4172 (3)	0.4357 (3)	0.0317 (8)	
H4	1.2586	0.3405	0.4931	0.038*	
C5	1.2143 (4)	0.5386 (4)	0.4468 (3)	0.0302 (8)	
C6	1.1591 (4)	0.6523 (3)	0.3620 (3)	0.0306 (8)	
H6	1.1576	0.7334	0.3702	0.037*	
C7	1.0414 (4)	0.7698 (3)	0.1692 (3)	0.0259 (7)	
H7A	0.9929	0.8481	0.2027	0.031*	
H7B	1.1349	0.7799	0.1128	0.031*	
C8	0.7481 (4)	0.7705 (3)	0.1797 (3)	0.0269 (8)	
H8	0.7560	0.6812	0.2263	0.032*	
C9	0.6954 (4)	0.8659 (4)	0.2601 (3)	0.0377 (9)	
H9A	0.6977	0.9525	0.2156	0.045*	
H9B	0.7751	0.8324	0.3168	0.045*	
C10	0.5210 (5)	0.8823 (4)	0.3222 (3)	0.0410 (10)	
H10A	0.5213	0.7973	0.3722	0.049*	
H10B	0.4908	0.9464	0.3705	0.049*	
C11	0.3920 (4)	0.9293 (3)	0.2392 (3)	0.0322 (8)	
H11	0.3880	1.0175	0.1918	0.039*	
C12	0.4406 (4)	0.8330 (4)	0.1607 (3)	0.0414 (10)	
H12A	0.3600	0.8662	0.1045	0.050*	
H12B	0.4380	0.7468	0.2062	0.050*	
C13	0.6155 (4)	0.8160 (4)	0.0976 (3)	0.0378 (9)	
H13A	0.6452	0.7509	0.0504	0.045*	
H13B	0.6143	0.9006	0.0463	0.045*	
C14	0.9912 (4)	0.6381 (3)	0.0655 (3)	0.0255 (7)	
H14	0.9030	0.6289	0.0329	0.031*	
C15	1.1251 (4)	0.6565 (4)	−0.0355 (3)	0.0296 (8)	
C16	1.0875 (4)	0.7752 (4)	−0.1185 (3)	0.0298 (8)	
C17	1.1986 (5)	0.7917 (4)	−0.2178 (3)	0.0367 (9)	
C18	1.3514 (5)	0.6900 (5)	−0.2301 (3)	0.0454 (11)	
H18	1.4274	0.7007	−0.2951	0.055*	
C19	1.3921 (5)	0.5718 (4)	−0.1457 (4)	0.0476 (11)	
H19	1.4954	0.5038	−0.1541	0.057*	
C20	1.2793 (4)	0.5554 (4)	−0.0496 (3)	0.0368 (9)	
H20	1.3068	0.4759	0.0063	0.044*	
C21	1.2282 (7)	0.9219 (5)	−0.4076 (3)	0.0797 (17)	
H21A	1.3418	0.9132	−0.4048	0.120*	
H21B	1.1715	1.0074	−0.4549	0.120*	
H21C	1.2288	0.8521	−0.4400	0.120*	

### Atomic displacement parameters (Å^2^)

**Table:**

	*U*^11^	*U*^22^	*U*^33^	*U*^12^	*U*^13^	*U*^23^
Br1	0.0577 (3)	0.0246 (2)	0.0575 (3)	−0.00942 (19)	−0.0134 (2)	−0.00831 (19)
Br2	0.0490 (3)	0.0547 (3)	0.0320 (2)	−0.0205 (2)	−0.01256 (18)	−0.00628 (18)
O1	0.0277 (14)	0.0408 (16)	0.0439 (16)	−0.0053 (13)	0.0025 (12)	−0.0038 (13)
O2	0.0370 (16)	0.0440 (16)	0.0302 (14)	−0.0040 (13)	0.0040 (12)	−0.0011 (12)
O3	0.0602 (19)	0.0570 (18)	0.0303 (15)	−0.0259 (16)	0.0092 (13)	−0.0038 (14)
N1	0.0405 (18)	0.0227 (15)	0.0294 (16)	−0.0106 (14)	−0.0055 (14)	−0.0073 (13)
N2	0.0216 (15)	0.0262 (15)	0.0263 (15)	−0.0077 (12)	−0.0019 (12)	−0.0068 (12)
C1	0.0208 (18)	0.0288 (19)	0.0291 (18)	−0.0088 (15)	−0.0031 (15)	−0.0048 (15)
C2	0.0186 (18)	0.0276 (18)	0.0313 (19)	−0.0066 (15)	0.0006 (14)	−0.0094 (15)
C3	0.028 (2)	0.0219 (18)	0.036 (2)	−0.0074 (16)	−0.0032 (16)	−0.0082 (16)
C4	0.027 (2)	0.029 (2)	0.031 (2)	−0.0038 (17)	−0.0074 (16)	−0.0005 (16)
C5	0.0239 (19)	0.039 (2)	0.0297 (19)	−0.0106 (17)	−0.0051 (15)	−0.0097 (17)
C6	0.0249 (19)	0.032 (2)	0.037 (2)	−0.0127 (16)	0.0005 (16)	−0.0098 (17)
C7	0.0232 (18)	0.0268 (18)	0.0297 (18)	−0.0096 (15)	−0.0042 (15)	−0.0073 (15)
C8	0.0257 (19)	0.0221 (17)	0.0311 (19)	−0.0081 (15)	−0.0016 (15)	−0.0047 (15)
C9	0.030 (2)	0.048 (2)	0.035 (2)	−0.0078 (18)	−0.0039 (17)	−0.0168 (18)
C10	0.036 (2)	0.048 (2)	0.032 (2)	−0.0031 (19)	−0.0032 (18)	−0.0155 (18)
C11	0.028 (2)	0.0266 (19)	0.036 (2)	−0.0076 (16)	0.0033 (16)	−0.0049 (16)
C12	0.027 (2)	0.050 (2)	0.050 (2)	−0.0111 (19)	−0.0040 (18)	−0.018 (2)
C13	0.027 (2)	0.054 (3)	0.039 (2)	−0.0119 (19)	−0.0026 (17)	−0.0247 (19)
C14	0.0221 (18)	0.0295 (19)	0.0270 (18)	−0.0080 (15)	−0.0046 (14)	−0.0098 (15)
C15	0.027 (2)	0.039 (2)	0.0253 (18)	−0.0134 (17)	−0.0032 (15)	−0.0096 (16)
C16	0.025 (2)	0.037 (2)	0.0259 (18)	−0.0096 (17)	−0.0037 (15)	−0.0072 (16)
C17	0.038 (2)	0.049 (2)	0.028 (2)	−0.020 (2)	0.0007 (17)	−0.0109 (18)
C18	0.035 (2)	0.073 (3)	0.037 (2)	−0.026 (2)	0.0066 (19)	−0.024 (2)
C19	0.026 (2)	0.062 (3)	0.054 (3)	−0.003 (2)	−0.001 (2)	−0.032 (2)
C20	0.026 (2)	0.042 (2)	0.041 (2)	−0.0053 (18)	−0.0057 (17)	−0.0149 (18)
C21	0.131 (5)	0.086 (4)	0.029 (2)	−0.063 (4)	0.027 (3)	−0.013 (2)

### Geometric parameters (Å, °)

**Table:**

Br1—C3	1.900 (3)	C9—C10	1.514 (5)
Br2—C5	1.895 (3)	C9—H9A	0.9700
O1—C11	1.423 (4)	C9—H9B	0.9700
O1—H1	0.8200	C10—C11	1.499 (5)
O2—C16	1.364 (4)	C10—H10A	0.9700
O2—H2	0.8200	C10—H10B	0.9700
O3—C17	1.363 (4)	C11—C12	1.509 (5)
O3—C21	1.416 (4)	C11—H11	0.9800
N1—C2	1.381 (4)	C12—C13	1.520 (5)
N1—C14	1.448 (4)	C12—H12A	0.9700
N1—H1A	0.8600	C12—H12B	0.9700
N2—C14	1.476 (4)	C13—H13A	0.9700
N2—C7	1.482 (4)	C13—H13B	0.9700
N2—C8	1.490 (4)	C14—C15	1.535 (4)
C1—C6	1.389 (4)	C14—H14	0.9800
C1—C2	1.396 (4)	C15—C16	1.385 (5)
C1—C7	1.517 (4)	C15—C20	1.388 (5)
C2—C3	1.395 (5)	C16—C17	1.397 (5)
C3—C4	1.376 (4)	C17—C18	1.381 (5)
C4—C5	1.376 (5)	C18—C19	1.391 (6)
C4—H4	0.9300	C18—H18	0.9300
C5—C6	1.374 (5)	C19—C20	1.379 (5)
C6—H6	0.9300	C19—H19	0.9300
C7—H7A	0.9700	C20—H20	0.9300
C7—H7B	0.9700	C21—H21A	0.9600
C8—C9	1.513 (5)	C21—H21B	0.9600
C8—C13	1.518 (4)	C21—H21C	0.9600
C8—H8	0.9800		
			
C11—O1—H1	109.5	H10A—C10—H10B	107.9
C16—O2—H2	109.5	O1—C11—C10	107.9 (3)
C17—O3—C21	117.3 (3)	O1—C11—C12	112.8 (3)
C2—N1—C14	120.0 (3)	C10—C11—C12	109.8 (3)
C2—N1—H1A	120.0	O1—C11—H11	108.8
C14—N1—H1A	120.0	C10—C11—H11	108.8
C14—N2—C7	107.0 (2)	C12—C11—H11	108.8
C14—N2—C8	112.1 (2)	C11—C12—C13	110.9 (3)
C7—N2—C8	116.3 (2)	C11—C12—H12A	109.5
C6—C1—C2	120.2 (3)	C13—C12—H12A	109.5
C6—C1—C7	121.4 (3)	C11—C12—H12B	109.5
C2—C1—C7	118.4 (3)	C13—C12—H12B	109.5
N1—C2—C3	121.7 (3)	H12A—C12—H12B	108.1
N1—C2—C1	120.4 (3)	C8—C13—C12	112.7 (3)
C3—C2—C1	118.0 (3)	C8—C13—H13A	109.1
C4—C3—C2	121.9 (3)	C12—C13—H13A	109.1
C4—C3—Br1	118.5 (3)	C8—C13—H13B	109.1
C2—C3—Br1	119.6 (3)	C12—C13—H13B	109.1
C5—C4—C3	118.9 (3)	H13A—C13—H13B	107.8
C5—C4—H4	120.6	N1—C14—N2	113.6 (3)
C3—C4—H4	120.6	N1—C14—C15	113.8 (3)
C6—C5—C4	121.1 (3)	N2—C14—C15	109.0 (3)
C6—C5—Br2	119.3 (3)	N1—C14—H14	106.7
C4—C5—Br2	119.6 (3)	N2—C14—H14	106.7
C5—C6—C1	119.9 (3)	C15—C14—H14	106.7
C5—C6—H6	120.0	C16—C15—C20	118.9 (3)
C1—C6—H6	120.0	C16—C15—C14	118.9 (3)
N2—C7—C1	111.6 (3)	C20—C15—C14	122.1 (3)
N2—C7—H7A	109.3	O2—C16—C15	122.2 (3)
C1—C7—H7A	109.3	O2—C16—C17	116.8 (3)
N2—C7—H7B	109.3	C15—C16—C17	121.0 (3)
C1—C7—H7B	109.3	O3—C17—C18	126.0 (3)
H7A—C7—H7B	108.0	O3—C17—C16	114.9 (3)
N2—C8—C9	112.9 (3)	C18—C17—C16	119.1 (4)
N2—C8—C13	108.8 (3)	C17—C18—C19	120.2 (4)
C9—C8—C13	109.3 (3)	C17—C18—H18	119.9
N2—C8—H8	108.6	C19—C18—H18	119.9
C9—C8—H8	108.6	C20—C19—C18	120.1 (4)
C13—C8—H8	108.6	C20—C19—H19	120.0
C8—C9—C10	112.0 (3)	C18—C19—H19	120.0
C8—C9—H9A	109.2	C19—C20—C15	120.6 (4)
C10—C9—H9A	109.2	C19—C20—H20	119.7
C8—C9—H9B	109.2	C15—C20—H20	119.7
C10—C9—H9B	109.2	O3—C21—H21A	109.5
H9A—C9—H9B	107.9	O3—C21—H21B	109.5
C11—C10—C9	112.0 (3)	H21A—C21—H21B	109.5
C11—C10—H10A	109.2	O3—C21—H21C	109.5
C9—C10—H10A	109.2	H21A—C21—H21C	109.5
C11—C10—H10B	109.2	H21B—C21—H21C	109.5
C9—C10—H10B	109.2		

### Hydrogen-bond geometry (Å, °)

**Table:**

*D*—H···*A*	*D*—H	H···*A*	*D*···*A*	*D*—H···*A*
O2—H2···N2	0.82	1.89	2.614 (3)	147
O1—H1···O3^i^	0.82	2.41	3.048 (4)	135
O1—H1···O2^i^	0.82	2.13	2.897 (4)	155

Symmetry codes: (i) −*x*+1, −*y*+2, −*z*.
